# Grains, Cereals, and Legumes: Implications in Glycemic Index and Perspectives

**DOI:** 10.3390/foods14234038

**Published:** 2025-11-25

**Authors:** Manish Kumar Singh, Hyeong Rok Yun, Jyotsna S. Ranbhise, Sunhee Han, Songhyun Ju, Salima Akter, Seung Geun Yeo, Sung Soo Kim, Insug Kang

**Affiliations:** 1 Department of Biochemistry and Molecular Biology, School of Medicine, Kyung Hee University, Seoul 02447, Republic of Korea; manishbiochem@gmail.com (M.K.S.);; 2 Biomedical Science Institute, Kyung Hee University, Seoul 02447, Republic of Korea; 3 Department of Biomedical Science, Graduate School, Kyung Hee University, Seoul 02447, Republic of Korea; 4 Department of Otorhinolaryngology—Head and Neck Surgery, College of Medicine, Kyung Hee University Medical Center, Kyung Hee University, Seoul 02453, Republic of Korea

**Keywords:** carbohydrates, starch, fiber, cereals, glycemic response

## Abstract

Background/Objectives: The glycemic index (GI) is a critical factor in managing blood sugar levels and related diseases. Grains, as staple foods consumed worldwide, are primary sources of carbohydrates, starch, and dietary fiber (DF). The carbohydrate composition of grains can significantly influence postprandial blood glucose levels. Therefore, understanding how different carbohydrate components affect blood glucose is essential. Methods: This study retrospectively examined the relationship between carbohydrate composition and GI in various grains, cereals, and legumes. Data on grain and cereal components were obtained from reputable public databases, including PubMed, the U.S. Department of Agriculture (USDA), FooDB, and published studies. Results: Analysis of the GI and glycemic load (GL) across grain components revealed several key findings. In addition to total carbohydrate (TC), both dietary starch (DS) and dietary fiber (DF) had substantial effects on GI. Interestingly, total sugar (TS), often considered a primary concern, showed no significant association with GI. Multiple regression and linear regression analyses demonstrated strong correlations between GI and both TC and DS. Among ratio metrics, the TC-to-DF ratio displayed significant correlation with GI (R = 0.48, *p* = 0.0003), followed by the DS-to-DF ratio (R = 0.33, *p* = 0.0159). The TS-to-DF ratio, however, showed no significant correlation (R = 0.04, *p* = 0.7544). Conclusions: These findings suggest that carbohydrate-to-fiber ratios, especially TC-to-DF, may play an important role in determining GI. Other dietary components, such as dietary fiber and dietary starch, might also affect these results. Additional studies are needed to examine how factors beyond carbohydrates influence GI. These observations may help guide future work aimed at better understanding dietary effects on health. Further, our results offer valuable insights for making healthier nutritional choices and improving the management of chronic diseases.

## 1. Introduction

Grains are staple foods worldwide and serve as the primary ingredients in bread, breakfast cereals, legumes, and grain-derived beverages. They are typically consumed either as whole grains (WGs) or refined grains (RGs). Refined grains undergo extensive processing steps, including decortication, dehulling, grinding, and milling. Numerous studies have shown that incorporating WGs into the diet provides significant health benefits compared to RGs. However, inconsistencies remain regarding grain classifications, particularly between gluten-free and gluten-containing varieties, due to the absence of a universally accepted definition of WGs. According to the 2010 Dietary Guidelines Technical Advisory Committee (DGTAC) for Americans, a whole grain must contain all three components of the original kernel: the bran (outer layer), germ (smallest part), and endosperm (main starchy core). Consumption of WGs has been linked to reduced risk of chronic diseases, improved nutrient intake, and enhanced overall diet quality [1,2]. WGs may also help regulate blood glucose levels, serum triglycerides, total cholesterol, and low-density lipoprotein (LDL). In contrast, low-fiber RGs offer fewer health benefits [3,4], and any metabolic improvements are often attributed more to reduced dietary fat than to the grains themselves. Additionally, concerns have been raised regarding the methods used to determine the postprandial blood glucose levels and GI, as uncertainties persist about which grain components such as sugar (hexoses), dietary fibers (DF), both soluble or insoluble, and polysaccharides, directly influence GI.

Cereal grains primarily consist of starch (65 to 70% of total weight), proteins (6 to 12%), and fats (1 to 5%), along with trace minerals, vitamins, and DFs. DF in grain is derived mainly from non-starch polysaccharides (NSPs). Insoluble NSPs function as effective laxatives, whereas soluble fibers, either viscous (gel-forming) or non-viscous, play key roles in reducing postprandial blood glucose levels [5]. β-glycans, a type of soluble NSP, may lower plasma cholesterol and reduce cardiovascular risk, although evidence remains mixed [6]. Supplementation with gel-forming fiber has shown potential to support modest weight loss and mitigate multiple cardiometabolic risk factors [7]. Compared with WGs, RGs lack the pericarp (seed coat), aleurone layer, and germ (fibers, micronutrients, fats, and proteins), leaving primarily the starchy endosperm. Epidemiological studies associate WGs consumption with a reduce risk of cardiovascular disease (CVD) and type 2 diabetes mellitus (T2DM) [8]. For instance, whole grain rice contains more fiber than white rice, promoting slower digestion and carbohydrate absorption [9].

Dietary carbohydrates are further classified by their digestibility in the small intestinal. Polysaccharides containing α-1,4 glucosidic linkages are effectively hydrolyzed by α-amylase. Some starches, such as those in instant potatoes, are digested and absorbed rapidly, producing a high glycemic response (GR) when consumed in amounts delivering 50 g of available carbohydrates. In contrast, the oligosaccharides (OSs) and NSPs resist digestion by α-amylase due to the absence of digestible monomers like glucose. Although OSs occur in low amounts in most grains, as seen in Belgian endive (chicory) or certain dairy by-products, they serve as prebiotic compounds that promote gut health [10].

Foods with low GI (typically ≤ 55) are digested and absorbed more slowly, resulting in a gradual rise in blood glucose [11]. Low GI foods reduce GRs and promote the formation of resistant starch (RS) [12]. RS supports appetite regulation and energy homeostasis by modulating gut hormones such as peptide YY and glucagon-like peptide-1 (GLP-1) [13,14]. A high-fiber diet enhances microbial production of short-chain fatty acids (SCFAs), which support lipid metabolism, reduce adiposity [15,16], improve glycemic control [17], enhance satiety, reduce appetite, and aid in weight management [18]. These findings underscore the systemic metabolic benefits of DFs beyond their gastrointestinal effects [19].

Recent advances have clarified interactions between grain components and the gut microbiome. Undigested carbohydrates are fermented by colonic microflora into SCFAs [20], particularly butyrate and propionate, which strengthen gut barrier function, decrease systemic endotoxemia and inflammation, and regulate glucose homeostasis [21,22]. Foods rich in insoluble NSPs are less fermentable than RSs but still promote laxation. Although RS enhances the production of specific SCFAs, the distinct roles of RS, NSPs, and starch in shaping SCFA profiles and colonic health remain unresolved. The purpose of our study is to refine the classification of grains based on their carbohydrate-to-fiber ratios, which appear strongly associated with GIs. Improving grain classification through this approach may yield a more reliable system for predicting GI than conventional metrics, while enhancing the categorization of grains according to their carbohydrate-to-fiber composition. Highlighting the contributions of specific carbohydrate components in grains is essential for optimizing personalized dietary recommendations and improving postprandial glucose responses and insulin sensitivity.

## 2. Materials and Methods

### 2.1. Grain Carbohydrate Content Data Collection

This study was based on publicly available secondary data sources. Grain selection considered multiple criteria, including global availability and consumption, nutritional value, carbohydrate and dietary fiber content, glycemic index, health benefits, and the potential to produce nutritious end products. Since grains are a major source of essential macronutrients particularly carbohydrates and polysaccharides such as starch and fibers, their effects on blood glucose response were a key focus. Accordingly, the study emphasized widely available, carbohydrate-rich grains commonly used in products such as bread, muffins, oats, and muesli bars. Each grain species includes numerous varieties that differ in nutritional composition and processing methods, such as drying, milling, fractionation, hydration, fermentation, extrusion, cooking, baking, frying, steaming, and freezing, etc. WGs retain the bran and germ and therefore provide significantly more dietary fiber than RGs. WG consumption is associated with improved bowel function, including increased fecal bulk, softer stool consistency, and reduced colonic transit time (CCT), which collectively help alleviate constipation. Grain selection also accounted for the presence of well-characterized bioactive compounds linked to health benefits, such as high antioxidant capacity, low glycemic index, elevated fiber content, and prebiotic properties that support gut microbiota diversity. Additionally, the nutritional profile of grains is influenced by genetic variation, environmental conditions, and climate—all of which are important considerations when selecting grain cultivars for dietary and functional applications.

Information on grain components, particularly carbohydrates and dietary fiber, was gathered from multiple reputable sources, including USDA Data Central, FooDB, the Korean Food Composition Database (KFCD), the European Food and Safety Authority, the World Health Organization (WHO) Nutrient Data Portal, Food Standards Australia-New Zealand, the Australian Food Composition Database (Release 2.0), Mattilsynet, MyFoodData, NutrientOptimiser, NatureClaim, Nutritionvalue, Food Struct, PubMed, and other open-access resources. For each grain, cereal, and legume, analytical values were extracted as average amounts per 100 g. Values for TC (cumulative amount of all sugar types), TS (including glucose, fructose, and sucrose), DS, and DF were compiled from these sources to ensure consistency and comparability.

### 2.2. Estimation of Glycemic Index (GI) and Glycemic Load (GL) of the Grains

The GI values for the selected grains were compiled from the scientific literature and publicly accessible databases [23,24]. The GI and the GL values for each grain were primarily obtained from food data sources, such as the NHS Foundation Trust, and other platforms that provide updated information. The GI values were originally measured using either glucose or white bread as reference. Notably, when white bread was used as the reference, the GI value was multiplied by 0.7 to standardize it in comparison to glucose. GL values were calculated by multiplying the amount of carbohydrate in a specified serving size by the GI of that food. Carbohydrate content was obtained from original studies or reliable food composition data sources [25,26]. Additional data sources included MyPlate.gov (USDA), GestationalDiabetic.com, UniversityHealthNews.com., NourishedByScience.com, Glycemic Index Guide, RedcliffeLabs.com, Newlifeobgyn.com, MSDManuals.com., Dasilvainstitute.com., the online Glycemic Index Database (www.gilisting.com), and the official GI website and databases (www.glycemicindex.com). All data were accessed between January and May 2025 (Table 1).

### 2.3. Estimation of Carbohydrate Content and Its Fiber Ratios

The grain samples were analyzed to determine their individual sugar components, including total carbohydrate and dietary fiber content. The available carbohydrate fraction was then calculated using the following formula [27,28,29]:

$$\mathrm{available}\;\mathrm{carbohydrate}\;\left(g\right)\;=\;\mathrm{total}\;\mathrm{carbohydrate}\;\left(g\right)\;-\;\mathrm{dietary}\;\mathrm{fiber}\;\left(g\right)$$

This value represents the net carbohydrate content and serves as a standard reference for determining the glycemic index (GI) of foods. A similar approach was applied to estimate the available total sugar and starch contents of individual grains, and these values were used to assess their correlation with GI and GL. Notably, grains contained significantly lower amounts of simple sugars primarily hexose monosaccharides compared to total carbohydrates and starch. This occasionally resulted in negative values when the traditional method was applied. Notably, these negative values do not indicate the presence of “negative sugar” in any particular grain; rather, they reflect the calculation in relation to dietary fiber content. Typically, grains exhibit higher levels of complex carbohydrates (including starch), resulting in positive values for most samples when compared to total sugar content. Nevertheless, a few grains still showed negative values due to their specific composition (Table 2).

To minimize such anomalies, calculating available content as a carbohydrates-to-fiber ratio eliminates negative outputs (Table 2) [30]. The total sugar content represents the sum of glucose, fructose, and sucrose, whereas the total carbohydrate content includes all quantified sugar forms [31,32,33]. The calculated carbohydrate-to-dietary fiber ratios (g/g) for the analyzed grains are presented in Table 2. This suggests that using a fiber-ratio–based approach provides a more reliable metric than the traditional subtraction method for determining available carbohydrate content and, consequently, more accurately estimating the GI of foods.

### 2.4. Venn Diagram

To compare the multiple components among grains, Venn diagrams were generated using the InteractiVenn v1.1 online tool [34]. Advanced Interactive Venn Diagrams for Scientific Research online program was access for analysis via www.interactivenn.net. The four grain groups, including GI, were analyzed simultaneously. Separate Venn diagrams were constructed to illustrate both total carbohydrate content and carbohydrate-to-fiber ratios.

### 2.5. Statistical Analyses

Statistical analyses were performed to evaluate correlations among the carbohydrate components of grains using Microsoft Excel and GraphPad Prism (version 10.4.1). To assess the key variables among grain components, Principal Component Analysis (PCA) was performed to reduce the variables dimensionality by GraphPad Prism. Parallel analysis was examined to find the relationships between each variable by comparing their eigenvalues obtained through random simulations. For PCA, the total number of variables and components evaluated was four, and component selection was guided by the parallel analysis criteria. Subsequently, multiple linear regression analysis was employed to assess the correlation between the grain contents and their corresponding GI and GL values. Pearson’s correlation coefficient (r) was calculated to quantify linear associations, and statistical significance was determined using two-tailed tests complemented by one-way ANOVA at a 95% confidence level. Statistical significance was defined as *p* < 0.05. Pearson’s correlation coefficient (r) was further applied to explore the pairwise relationships between GI, GL, and individual grain constituents. The correlation coefficient (R) and corresponding regression line were derived from the best-fit estimates of the slope and intercept. Correlation strength was classified as weak (R < 0.4), moderate (0.4 ≤ R ≤ 0.6), or strong (R > 0.6). The values assigned for GI and carbohydrate content were calculated using standard biostatistical methods, and the results are presented as mean ± standard deviation (SD).

## 3. Results

The primary objective of this study was to identify the key carbohydrate components that significantly influence the GI of grains. Many grains are readily available as flour and serve as staple ingredients in numerous cuisines. The carbohydrate content of grains (*n* = 52) was analyzed and categorized as TC (g), DF (g), and TS (g) (Table 1). However, individual hexoses, such as glucose, fructose, and maltose, were not estimated or quantified for most grains, potentially leading to misclassification of foods that are high in WG content. According to the Whole Grains Council (WGC), refined-grain foods are defined as grain- or flour-based products that do not meet the criteria for classification as whole grains. In this study, grains were further categorized based on the grain content-to-fiber ratio to assess their effects on GI and GL, as presented in Table 2.

Comparative studies have demonstrated that the content-to-DF ratios provide more reliable and precise information than simply calculating the absolute difference between TC and DF. It represents a better approach than traditional methods and provides a clearer understanding of how DF contributes to TC content. It also influences the presence of other forms of sugar, which may affect glycemic response (GR), insulin sensitivity, and glucose levels [30]. This metric is also useful for gaining insights into the physiological relevance of WGs and RGs. It aids in identifying specific grains that minimize blood glucose spikes while providing high nutritional value and a lower glycemic response. The study considered four different components including TC, DS, DF, and TS and analyzed their correlation with GI and GL.

**Figure 1:**
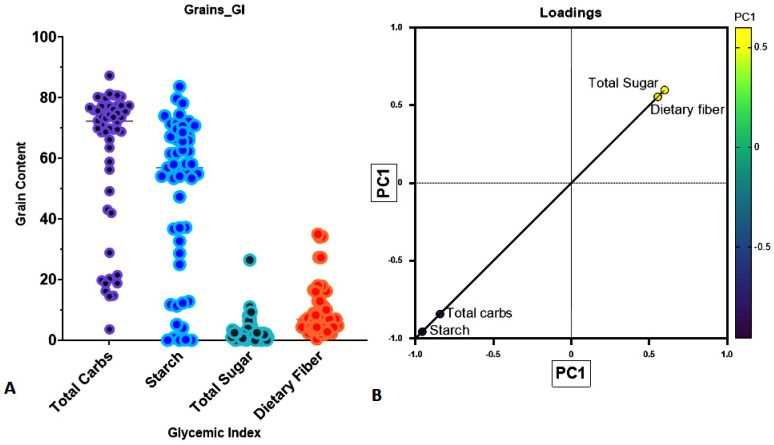
(**A**,**B**): PCA in different grain contents (TC, TS, DS, and DF with GI). The correlation plot illustrates the relationship between GI and grain carbohydrate contents in multiple grains (*n* = 52). (**A**) The graphs display the correlation between individual carbohydrate contents and GI. Each data point represents an individual grain, with their carbohydrate values plotted on the *Y*-axis and GI on the *X*-axis. (**B**) The image represents the loading vectors and PC scores of components. TC and DS group together, while TS and DF are clustered together.

Initially, a PCA analysis was performed to reduce the variability in grain components. Results revealed that TC was the most influential variable, accounting for 57.27% of the total variance, followed by DS (22.08%), TS (18.98%), and DF (1.68%), respectively (Table 3). Notably, the cumulative contribution of TC, DS, and TS exceeded the upper limit (95th percentile) of eigenvalues. TC and the DS were positively correlated, while TS and DF clustered together along the first principal component (PC1) (Figure 1A,B). These results indicate that TC and DS (loading scores of 0.310 and 0.399, respectively) were more influential variables than TS and DF (loading scores of 0.156 and 0.134, respectively). Collectively, TC and DS accounted for 79.34% of the total variance (Table 3), suggesting that TC and DS are key players in determining GI in grains.

**Figure 2:**
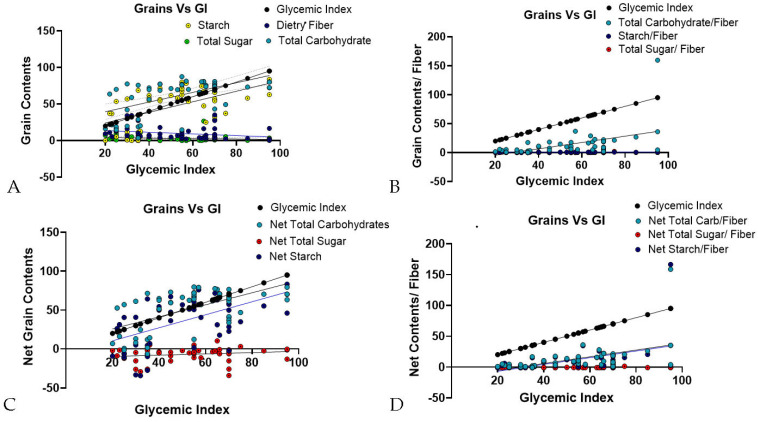
(**A**–**D**): The graphs represent MLRA in different grain contents (*n* = 52). The correlation plot illustrates the relationship between GI and grain carbohydrate contents. (**A**) MLRA between TC, TS, DF, and DS to GI. (**B**) MLRA between grain content-to-fiber ratios to GI. (**C**). MLRA between available (net) content in grains and to GI. (**D**). MLRA between available (net) content-to-fiber ratio in grains and to GI. Each data point represents an individual grain, with their carbohydrate content values plotted on the *Y*-axis and GI on the *X*-axis.

A correlation analysis was conducted to examine the correlation between individual grain components to GI and GL through multiple linear regression analysis (MLRA). The results showed that TC and DS had a strong and significant correlation with GI, with a highly significant coefficient—r- and *p*-values (Table 4). In contrast, TS and DF displayed weaker, negative correlations, supported by lower statistical r- and *p*-values (Figure 2A; Table 4). The graph analysis showed TC and DS values were closely clustered around the GI linear regression line, while TS and DF data points were more widely distributed, often near the *X*-axis, indicating weaker correlations. When analyzing the content-to-fiber ratios, the TC-to-DF and DS-to-DF ratios exhibited significant relationships to GI, while the TS-to-DF ratio did not exhibit a significant correlation (Figure 2B). The corresponding statistical coefficient (r) and *p*-values are represented in Table 4.

Intriguingly, these findings were compared with net carbohydrate contents using a conventional method. The available (net) carbohydrate, sugar, and starch contents were estimated in various grains using the standard conventional method (Table 2). The results indicate that the available carbohydrate content varies among different grains, aligning with expectations for those high in carbohydrates. However, this method appears to be less effective for grains with low carbohydrate content. Specifically, when applying the same formula to estimate net sugar content, the obtained values were negative for several grains, which highlights a limitation of the method. These findings suggest that while the conventional calculations are reliable for high-carbohydrate grains, they may not yield accurate results for grains with low carbohydrate content. A correlation analysis between available carbohydrate content and GI for each grain revealed a significant correlation between available carbohydrates and available starch but not for total sugar (Figure 2C; Table 5). Moreover, ratios of net grain content-to-fiber supported a strong correlation for both net TC and DS, while TS did not show a significant relationship (Figure 2D; Table 5). Overall, these findings suggest that TC and DS are critical variables of grains, while TS has minimal impact.

In addition to GI, GL exhibits a vital role in glycemic response (GR), as it reflects both GI of a food and the serving size consumed per meal, summarizing the cumulative GR over multiple intake events [35,36]. Thus, we evaluated the correlations between grain carbohydrates and GL. Our results showed that both TC and DS exhibited strong correlations with GL and significant r- and *p*-values (Figure S1A, Table S1). In contrast, DS and DF showed weaker, negative correlations, with lower statistical significance (Table S1). The data points for TC and DS were clustered around the GL linear regression line, while those for TS and DF were more widely placed. When analyzing the ratio of grain content-to-fiber ratio to GL, only the TC-to-DF ratio exhibited a significant correlation to GL, while the TS-to-DF and DS-to-DF ratios did not (Figure S1B; Table S1). Net carbohydrate analysis confirmed these findings—net TC and net DS both were significantly correlated with GL, while TS remained non-significantly related to GL (Figure S1C; Table S1).

The net grain content-to-fiber ratios were also evaluated. The net TC-to-DF ratio showed the most significant correlation with statistically significant r- and *p*-values, whereas net DS and TS did not exhibit a strong correlation (Table S2). In contrast, grains with higher GL and net sugar-to-DF ratios were widely dispersed from the regression line, with their corresponding data points positioned near the *X*-axis (Figure S1D; Table S2). In summary, the results indicated that both TC and DS exhibited strong correlation with GL, with data points predominantly clustered around the regression line near the *Y*-axis.

**Figure 3:**
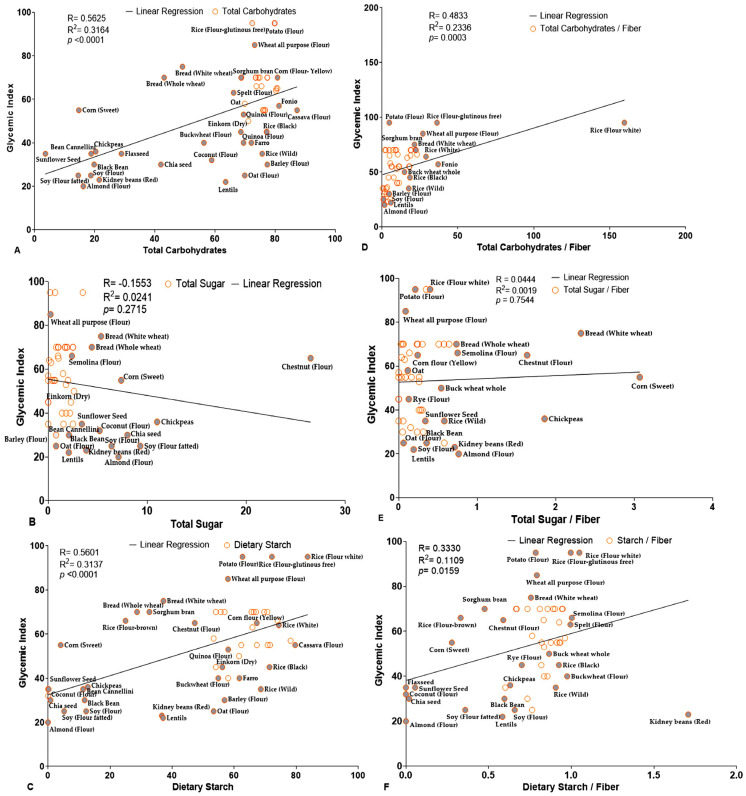
(**A**–**F**): Correlation plot illustrating the relationship between GI and carbohydrate content as well as carbohydrate-to-DF ratios in various grains (*n* = 52). (**A**–**C**) show the correlations between GI and a specific type of carbohydrate component. (**D**–**F**) depict the correlations between GI and corresponding carbohydrate content-to-DF ratios. In all plots, each data point represents distinct grain sample, with GI values on the *Y*-axis and carbohydrate metrics on the *X*-axis. Solid lines indicate linear regression trends, reflecting the strength and direction of associations. Selected data points are labeled in gray for emphasis and identification.

**Figure 4:**
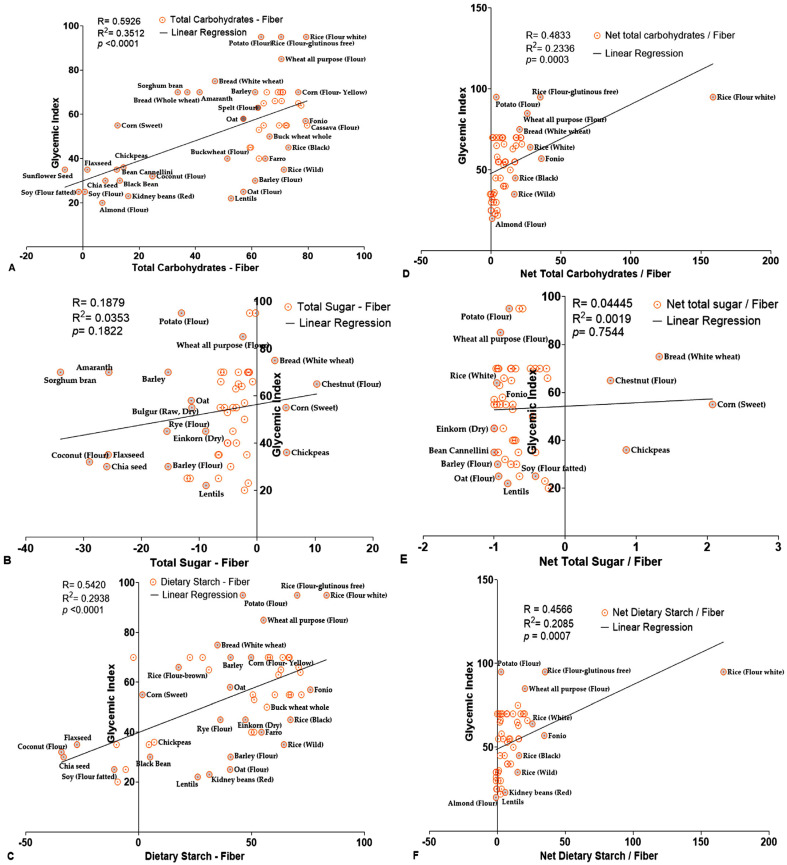
(**A**–**F**): Correlation plots illustrating the relationship between GI and net carbohydrate content as well as net carbohydrate content-to-DF ratios in various grains (*n* = 52). (**A**–**C**) display the correlations between GI and individual net carbohydrate contents. (**D**–**F**) display the correlation between GI and corresponding net carbohydrate content-to-DF ratios. Each data point represents a distinct grain sample, with GI values on the *Y*-axis and either net grain content or net carbohydrate-to-DF ratios on the *X*-axis. Solid lines indicate linear regression trends, representing the direction and strength of the associations. Labeled data points are highlighted in gray for emphasis and identification.

To further validate these findings, we conducted Pearson regression analyses of TC, TS, and DS with GI. TC and DS both showed strong and statistically significant correlations with GI (Figure 3A,C, Table 6). Graphical analysis revealed that various grains, such as soy flour, black bean, kidney bean, fatted soy flour, sunflower seed, corn seeds, chia seeds, and almond flour, were closely clustered around the regression line and *Y*-axis for both TC and DS. Additionally, the TC-to-DF and DS-to-DF ratios also showed significant correlation with GI, although the Pearson coefficient r- and *p*-values were slightly lower (Figure 3D,F, Table 6). Conversely, TS showed a negative and non-significant correlation with GI, both independently and as a ratio to fiber (Figure 3B,E, Table 6). These analyses oppose the traditional assumption that sugar is a primary factor for determining GI and GL. Further analyses of net carbohydrate content reinforced that both net TC and net DS were strongly correlated with GI, with highly significant coefficients r- and *p*-values (Figure 4A,C, Table 7). Their net content-to-fiber ratios particularly net TC and net DS exhibited a significant correlation with GI, with statistically significant r- and *p*-values (Figure 4D,F, Table 7). Conversely, the available TS did not show any significant relationships in either case (Figure 4B,E).

Since multiple regression analysis showed strong correlations of TC and DS with GL; therefore, further validation was evaluated using Pearson regression analyses. The pattern mirrored those observed in the GI analysis. Again, TC and DS showed strong correlation with GL, with statistically significant r- and *p*-values (Figure S2A,C, Table S3). Graphical analysis revealed that grains, such as soy flour, defatted soy flour, sunflower seed, sweet corn, black bean, chia seed, flaxseed, and almond flour, were closely associated with GL in both TC and DS. These grains aligned closely to the regression line and *Y*-axis, indicating that the incorporation of these grains, even in higher quantity in daily diets, may contribute to a slower blood glucose response, thus preventing the high risk of T2D. However, the available total sugar failed to show any significant relationships in either case (Figure S2B, Table S3).

The TC-to-DF ratio showed significant correlation to GL, with statistically significant r- and *p*-values (Figure S2D,F, Table S3), whereas the DS-to-DF ratio was unable to reach the statistical significance, yielding lower r- and *p*-values (Figure S2E, Table S3). Additionally, TS exhibited a negative and non-significant correlation with GL, both independently and when analyzed as a ratio to fiber. Net TC and net DS continued to show a strong correlation with GL, with significant r- and *p*-values (Figure S3A,C, Table S4). The net content of grains and their ratios to DF displayed a significant correlation with net TC and net DS, with significant statistical r and *p*. However, net TS did not show a significant relationship in either case (Figure S3B,E, Table S4).

**Figure 5:**
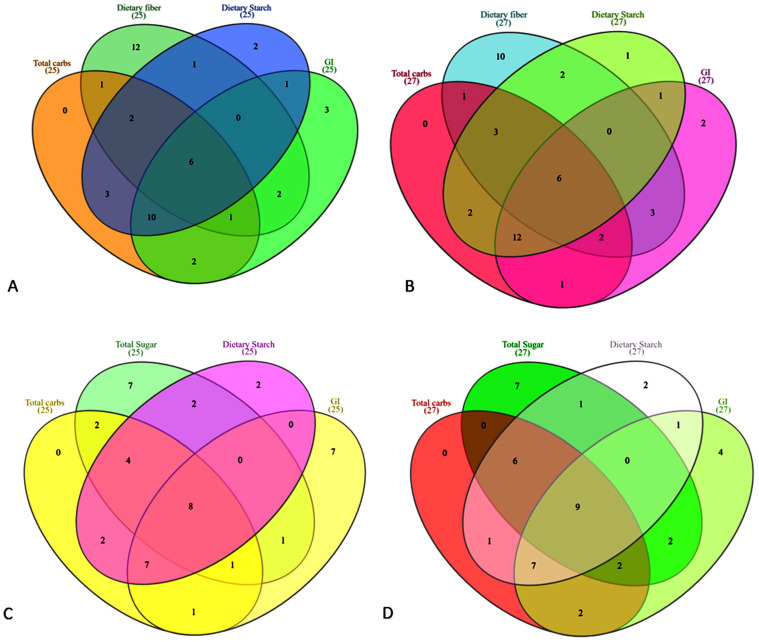
(**A**–**D**): Venn diagrams illustrating the distribution of common grains across carbohydrate categories. (**A**) Total carbohydrates with low sugar content. (**B**) Total carbohydrates with high sugar content. (**C**) Carbohydrate-to-fiber ratios with low sugar content. (**D**) Carbohydrate-to-fiber ratios with high sugar content. Numbers indicate the number of grain varieties analyzed and the overlapping grains shared among groups.

We analyzed the distribution of common grains across various groups based on their minimum and maximum carbohydrate contents to identify those with the lowest and highest levels. Four groups were evaluated, along with three forms of sugars in different grain categories and their GI. The results were illustrated using Venn diagrams (Figure 5A–D). In the normal carbohydrate category, sweet corn, cannellini beans, black beans, chickpeas, red kidney beans, and buckwheat flour consistently showed the lowest carbohydrate contents. Conversely, sorghum whole grain, white corn flour, barley flour, sorghum flour, potato, and cassava flour exhibited the highest values across TC, DF, DS, and GI (Figure 5A,B). Similarly, for the carbohydrate-to-fiber ratio, flaxseed, chia seeds, coconut flour, cannellini beans, rye flour, barley flour, oat flour, and lentils showed the lowest ratios. In contrast, sorghum whole grain, cassava flour, yellow corn flour, white sorghum grain, white sorghum flour, millet, semolina flour, glutinous rice flour, and white rice flour had the highest ratios (Figure 5C,D). These findings suggest that consuming low-carbohydrate grains may help reduce GI, while limiting the intake of high-carbohydrate grains could aid in GI management. Similarly, grains with a low carbohydrate-to-fiber ratio are likely to provide metabolic benefits, whereas those with a high ratio may have adverse effects if consumed in large quantities.

We also conducted an analysis to identify any outliers based on TC, TS, and DF content in gains. The data displayed that eleven grains were TC outliers such as black bean, bean cannellini, chia seed, chickpeas, sweet corn, flax seed, almond flour, soy flour, soy flour (fatted), kidney beans (red), and sunflower seed; six were TS outliers (chia seed, chickpeas, sweet corn, almond flour, chestnut flour, and soy flour (fatted)), and five were DF outliers such as amaranth, chia seed, flaxseed, coconut flour, and sorghum bran. Despite being outliers, these grains are extensively consumed and included in various grain-based meals, and thus are not excluded from the analyses.

Overall, the results showed that TC and DS are the most critical determinants of GI, whereas TS (with or without net content) contributes minimally. Notably, DF appears to play a central role in moderating these relationships and may enhance the predictive value of TC and DS for both GI and GL.

## 4. Discussion

The relationship between carbohydrate composition and dietary fiber and other aspects of carbohydrate quality, such as GI and GL, remains unknown. However, an important question arises: which form and quantity of grains are most beneficial, particularly concerning postprandial glucose response? The nutritional content of grains can vary significantly due to the processing methods such as grinding, germination, and drying. For instance, WG rice has been shown to significantly reduce the blood glucose area under the curve (AUC) compared to its refined counterpart [37]. Conversely, wheat and rye do not show a significant reduction in postprandial blood glucose levels when compared to their refined forms [38]. Nevertheless, these grains have been found to significantly lower plasma cholesterol levels [39]. In this context, two prevailing hypotheses suggest that individual components of WG (e.g., fiber and starch) affect disease-related biomarkers, including plasma cholesterol and insulin secretion. Despite this, the overall health benefits of WGs appear to exceed the sum of their individual components. It is well established that refining processes remove many health-promoting components naturally found in WGs. WG and RG cereals differ in their content of TC, DS, and TS, all of which influence blood glucose levels. Thus, it is crucial to determine which key components actually impact most to GI in foods.

GI plays a significant role in maintaining physiological homeostasis, especially in managing chronic diseases. Research suggests that the proportion of carbohydrates and DF is crucial for assessing food quality. Diets rich in DF and low in carbohydrates are associated with reduced risk of chronic diseases [27]. However, accurately determining GI of a food remains challenging. Traditionally, GI is calculated based on the available carbohydrates derived by subtracting DF from TC, and then comparing the AUC to that of a reference carbohydrate, such as glucose or white bread [40]. The carbohydrate content in each grain can vary due to several factors, including the processing methods used to prepare refined flour, grinding, rolling, and the particle size used during processing. Processing and preparation of grains alter their nutritional value, most notably by changing the characteristics of starch. For instance, milling breaks down the cell walls of grain, increasing bio-accessibility of starch to digestive enzymes. Additionally, cooking or streaming allows water to be absorbed by starch granules, making them easier to digest through enzymatic degradation. Any remaining undigested starch is fermented by gut microbiota in the large intestine [41].

In this study, we analyzed the grain-based carbohydrates, including TC, TS, and DS, and examined their ratios to DF. We then compared these ratios to available carbohydrate (net) contents. Results indicated that the available carbohydrate and their ratios to DF offer a more reliable predictor of GI than only measuring available contents. Previous findings have shown that this ratio correlates more positively with GI across various fruits [30]. Notably, both TC and dietary starch, along with their ratios to DF, showed a stronger correlation with GI than total sugar and their respective ratios to fiber. These results underscore the significant role of the fibers in modulating the GI, especially in grains. While TC was significantly correlated with GI, the grains were widely spread along the regression curve, indicating possibilities of additional factors. In contrast, the TC-to-DF ratio displayed a closer association with GI compared to other types of sugars, with data points aligning more closely with the regression line, despite a slightly lower correlation coefficient. For instance, grains such as millet, fonio, sorghum flour, semolina flour, and glutinous rice flour deviated from the regression curve when only total carbohydrate content was considered. However, when the TC-to-DF ratio was included, these grains aligned more closely with the regression curve. This indicates that fiber is a more significant determining factor than simply subtracting it from TC, which showed a weaker correlation and lower significant values. A similar pattern emerged when available carbohydrates were correlated with GI, showing consistency with TC analysis. The spatial distribution of grains remained largely consistent, with a few exceptions, such as sunflower seeds and fatted soy flour. Notably, fiber ratio showed stronger correlation across the various grains. The results indicated that incorporating fiber ratio exhibited more comparable trends, reinforcing the central role of DF in determining GI.

In contrast, TS content exhibited a weak and statistically nonsignificant correlation with GI. However, when we evaluated the TS-to-DF ratio, both the correlation and the regression curve improved. Despite this enhancement, the correlation remained negative, and the grain data points remained widely scattered around the regression line. This scattering is attributed to the less sugar content in grains, where the difference between sugar and fiber resulted in negative values (indicating negligible sugar content), making them safer for consumption. Notably, the TS-to-DF ratio showed a stronger relationship with GI than the net TS-to-DF ratio. When we analyzed DS and their ratio to fiber, DS displayed a better correlation with GI compared to TS. The relationship was further enhanced when the starch-to-fiber ratio was applied, resulting in a tighter clustering of data points along the regression line. Available starch also showed a significant correlation with GI; however, the distribution of certain grains, such as flaxseed, coconut flour, chia seed, defatted soy flour, and almond flour, was positioned on the negative *X*-axis. This correlation improved when a ratio parameter (DS-to-DF) was introduced, causing those data points to align more closely with the regression line. The analysis indicated that the fiber ratio demonstrated a stronger correlation with GI compared to the conventional method for determining the net amount of all forms of sugar and its correlation with GI.

The standard method for estimating available carbohydrates for GI involves subtracting DF content from TC (TC-DF). However, our findings indicate that this approach may lead to significant inaccuracies when used as a reference for GI determination. For instance, in soy flour, the difference between TC and DF was 0.70 g, which corresponds to a TC/DF ratio of 1.039. Using this difference as the reference value for GI, prediction suggests that there would be a minimal increase in postprandial blood glucose levels. In contrast, cassava flour had a difference of 79.64 g, corresponding to a TC/DF ratio of 11.397. Employing the difference method predicts a significant rise in glycemic response, whereas the ratio-based approach indicates a lower glycemic impact. A similar pattern was observed in certain millet varieties, where the TC–DF difference was 71.07 g, and the TC/DF ratio was 22.34. In this case, the ratio aligned more closely with the observed GI curve than the subtraction method. Overall, these findings suggest that the fiber ratio matrix (TC/DF) serves as a more accurate and reliable predictor of glycemic response than the conventional subtraction method. Relying on the subtraction method may lead to miscalculations of the available carbohydrate content and, consequently, misinterpretation of GI values. Therefore, the ratio may serve as a standard metric for assessing carbohydrate quality in GI prediction, given its stronger correlation with observed postprandial glycemic patterns compared to the subtraction-based approach.

GL is another crucial metric that reflects the postprandial blood glucose response. GL calculation is based on available carbohydrate per serving as the following: GL = (carbohydrate content of each food item) × (number of serving/d) × (GI) [36]. GL has shown a stronger association with plasma lipid levels than with insulin response or glycemic fluctuations [42,43]. The introduction of the GL concept allows a comparison to be made between the probable glycemic effect of realistic portions of foods that contain different amounts of carbohydrates. However, the utility of both GI and GL has been debated due to several limitations, including their inability to fully account for insulin response [44], high intra- and inter-subject variability in GR [45], and reduced accuracy when foods are consumed in mixed meals [46]. GL is typically classified as low (≤10), medium (>10–<20), or high (≥20). The relationship between GI and GL is not always linear or straightforward. For instance, a high-GI food can have a low GL if consumed in small quantities, while a low-GI food may result in a high GL depending on the serving size. Notably, a low-GL diet can be achieved by selecting small portions of carbohydrate-rich foods, which provides a more practical strategy for managing postprandial blood glucose levels [47].

In this analysis, many grains showed combinations of high GI but low GL. For instance, black bean (GI: 30; GL: 2); flaxseed (GI: 35; GL: 0.6); soy flour (GI: 25; GL: 4.5); lentils (GI: 22; GL: 3); brown rice (GI: 55; GL: 18); and white rice (GI: 64; GL: 26) illustrated that even foods with relatively high GI can exhibit low GLs. Among the components of the grain that were examined, TC showed a significantly stronger correlation with GL than TS, which did not reach statistical significance in the analysis. Understanding these relationships is crucial for developing dietary strategies that optimize GR, particularly for individuals with nutrient-related metabolic disorders. Evidence from several randomized controlled trials indicates that dietary patterns characterized by low GI and GL may contribute to weight loss, improved insulin sensitivity and reduced cardiovascular risk factors [48].

Grains are rich in polysaccharides (DS and DF), which contribute to carbohydrate digestion and absorption. Soluble and insoluble forms of non-digestible carbohydrates are found in various grains. Notably, soluble DF binds to glucose and form a physical barrier that delays absorption, significantly affecting postprandial blood glucose levels [49,50]. An increased intake of soluble DF has been associated with significant glycemic control in diabetic patients. For instance, a diet rich in oats has been linked with significantly lower glycated hemoglobin and fasting plasma glucose levels in diabetic patients. Oat flour exhibits a lower GI and GL (GI: 25 and GL: 3) compared to whole oats (GI: 58 and GL: 16), indicating that oatmeal may have a more pronounced effect than a typical cereal-based diet [51,52]. This synergistic effect underscores the importance of soluble DF in regulating blood glucose levels and consequently GI [53]. Studies have shown that consuming low-GI foods such as psyllium, guar, oatmeal, barley, and legumes are effective not only in controlling appetite and managing body weight but also in reducing the risk of chronic diseases, especially T2DM, CVD, and possibly certain types of cancer [29].

Numerous studies have explored the role of DF in glucose metabolism, however, limited, and inconclusive evidence exists regarding its specific influence on carbohydrate digestion and absorption. DFs primarily exist in two forms: soluble DF (SDF) and insoluble DF (IDF) [54]. SDF can be further classified into two types: soluble viscous fibers (e.g., psyllium husk) and soluble non-viscous fibers (e.g., inulin, guar gum, wheat dextrin, or acacia). These DFs influence glucose absorption and consequently the GI of foods [6]. Recent studies have reported that viscosity of fiber correlates with postprandial plasma glucose levels due to its ability to delay gastric emptying and glucose absorption in the small intestine [5,50]. The proposed mechanism suggests that viscous soluble fibers slow gastric emptying and facilitating nutrient delivery to the distal ileum. This process stimulates the release of glucagon-like peptide-1 (GLP-1), which reduces food intake, enhances glucose-dependent insulin secretion, decreases glucagon secretion, promotes pancreatic β-cell growth, improves insulin production and sensitivity, and prolongs gastric emptying [55,56,57]. Fermentation of SDF by gut microbes produces SCFAs such as butyrate, acetate, and propionate. These metabolites support the growth of beneficial colonic bacteria [58,59] and exhibit anti-inflammatory and anti-oxidant properties, while also enhancing insulin sensitivity [60]. In contrast, IDFs act mainly as bulking agents and natural laxatives, increasing fecal mass and reducing intestinal transit time [61]. Despite these insights, comprehensive data on the interactions between various fiber types, starch fractions, and their effects on glucose and carbohydrates metabolism across various grains remain limited. Ancient grains such as spelt, einkorn, and emmer possess higher dietary fiber content compared to certain modern wheat varieties, thereby enhancing their overall nutritional profile [62]. Additionally, grains including chia, barley, millet, Khorasan wheat, buckwheat, and brown rice have been associated with favorable health outcomes in individuals with diabetes [63]. The present analysis further revealed that einkorn, chia seed, barley (in both raw and flour forms), buckwheat, and brown rice exhibit a low TC-to-DF ratio and a significant correlation with GI. These findings suggest that increasing the dietary proportion of such grains may contribute to improved glycemic response and enhanced insulin sensitivity.

The dietary contents in WG and its refined counterparts such as flour and broken cereals showed significant differences due to processing methods such as grinding, germination, and drying, which can affect the nutritional value and thereby impact the GI of cereals. For instance, barley and oats are particularly rich in both soluble and insoluble DFs, with fiber content ranging from 10 to 28% [64,65] and 10–38% [66] (on a dry matter basis), respectively. Rye also serves as excellent source of DF, with contents ranging from 14 to 21% on a dry weight basis [67]. Most oat- and barley-based foods are high in DF, nutrient-dense, and satiating, making them an ideal choice for weight management and reducing BMI [68]. The U.S. Food and drug Administration (FDA) has approved a health claim that SDF from oats may reduce the risk of heart diseases. Wheat contains 9–20% DF (on a dry weight basis) and includes important components such as arabinoxylan (AX) and β-D-glucan. Grains like amaranth, quinoa, and buckwheat are naturally gluten-free and rich in DF. Amaranth contains 9 to 21%, quinoa 7 to 21%, and buckwheat 7 to 11.9% [69]. In this analysis, flaxseed, amaranth, chia seed, coconut flour, and sorghum bran were found to have high dietary fiber content. Moreover, the ratio of TC-to-DF showed a stronger correlation with GI. These findings suggest that high-fiber grains may play a crucial role in regulating blood sugar levels. However, further molecular evidence is needed to confirm these effects through both in vivo and in vitro studies. Incorporating these grains into the regular diets may offer health benefits that go beyond glucose regulation, ultimately contributing to overall well-being.

Despite the numerous health benefits associated with WG intake, inconsistencies persist due to variations in study design, population selection, and the types and quantities of grains included in a diet. Notably, low-GI foods may reduce the risk of diabetes by decreasing oxidative stress, as indicated by the urinary excretion of the oxidative stress marker 8-hydroxy prostaglandin F2α [70]. Elevated levels of ROS are considered key contributors to hyperglycemia-induced complications in diabetes, including insulin resistance, dyslipidemia, and β-cell dysfunction—all of which impair glucose tolerance [71]. A portfolio diet, where food intake is tailored to include components known to reduce CVD risk, has demonstrated beneficial effects on biomarkers such as C-reactive protein (CRP) and LDL cholesterol [72]. Our findings suggest that gains such as whole wheat flour, white rice, brown rice, cereals, and pulses, which are typically high in dietary fiber and relatively low in available carbohydrates, may have a more favorable effect on GI. A study indicated that a diet based on brown rice significantly improved bowel function compared to diets based on white rice or wheat, likely due to its higher content of insoluble dietary fiber [73]. Furthermore, evidence shows that consuming bread made from mixed white wheat or rye flour, supplemented with berries, leads to a substantial reduction in postprandial insulin responses, thereby lowering the overall GI [74]. These findings suggest that incorporating fruits into grain-based foods may enhance insulin sensitivity and improve GR, potentially mitigating negative metabolic outcomes.

In addition to dietary modifications, regular exercise and continuous blood glucose monitoring are crucial for reducing the risk of chronic disease. Over the past decade, real-time continuous glucose monitoring (RT-CGM) and flash or intermittently scanned continuous glucose monitoring (FGM or isCGM), which measure interstitial glucose levels using subcutaneous sensors, have proven effective in lowering hemoglobin A1c (HbA1c) levels over specified periods [75,76]. HbA1c remains a key biomarker for diagnosing and managing diabetes and insulin resistance. A recent advance in this area is the “WaveFlex” optical fiber biosensor, based on localized surface plasmon resonance (LSPR). Fabricated using an S-tapered, waist-expanded fiber design and incorporating AuNPs, CeO_2_ nanorods, and WS_2_ nanosheets, this sensor exhibits a broad linear detection range of 0–1000 µg/mL [77]. Monitoring blood glucose and HbA1c responses to specific grain-based diets further enables personalized grain selection based on individual glycemic profiles [78].

Photoplethysmography (PPG), which detects blood volume changes using light, has emerged as a promising non-invasive technique for glucose monitoring. Recent studies have demonstrated that deep neural networks (DNNs) can accurately predict blood glucose levels (BGLs) from PPG signals [79]. The integration of artificial intelligence (AI) and machine learning (ML) further improve the accuracy and stability of PPG-based BGM [80,81]. Non-invasive approaches using electromagnetic ring resonators measure glucose by sensing changes in tissue dielectric properties. Future research should focus on improving glucose selectivity by reducing interference from other blood constituents and developing circuit designs that accommodate diverse tissue volumes to enhance measurement robustness [82].

Glucose oxidase (GOx)-based biosensors employ enzyme-modified nanomaterials such as carbon nanotubes, graphene, and carbon aerogels for highly sensitive electrochemical glucose detection. However, challenges related to immunogenicity, long-term stability, and biocompatibility still limit large-scale commercialization [83]. Addressing these issues, along with adequate training for clinical staff, is essential for reliable use. Self-monitoring remains central to diabetes management, and CGM technologies further support personalized nutrition by revealing postprandial glycemic responses to grain-based foods. This individualized feedback helps users assess the metabolic impact of different grains. Continued research is needed to better understand how grain consumption patterns influence metabolic health.

## 5. Conclusions

Numerous epidemiological studies suggest that higher intake of whole grains (WGs) and WG-based foods may be associated with reduced risks of chronic diseases, including CVD [84,85], T2DM [86,87], certain cancers [88,89,90], and other non-communicable chronic conditions [86,89]. Our study found a significant correlation between TC and DS with both GI and GL. Notably, the TC-to-DF ratio showed a more significant correlation with both GI and GL. Given that WGs provide higher levels of micronutrients, phytochemicals, and DF, they may contribute to improved diet quality [91,92]. The GI of foods is influenced not only by carbohydrate content but also by factors such as polysaccharides, fiber structure, particle size, micronutrients, fats, protein, minerals, and cooking methods [93].

These findings suggest that the carbohydrate-to-DF ratio could serve as a promising indicator for evaluating grain types and portion sizes, especially for individuals managing diabetes or body weight. Although long-term dietary patterns rich in WGs, cereals, legumes, and nuts have been associated with potential health benefits, the extent to which these patterns influence life expectancy remains uncertain. Emerging evidence suggests that adopting such practices earlier in life may offer greater benefits than initiating them later, though this requires further confirmation [94]. Given these uncertainties, our findings highlight the need for additional exploratory work on how grain characteristics and processing methods such as grinding, germination, or the use of intact versus refined forms may influence metabolic outcomes. Comparative investigations into WGs versus RGs, particularly their effects on insulin responses and blood glucose levels, could provide clearer insight into the potential role of whole and mixed grains in supporting healthier dietary patterns.

### Limitations and Future Perspectives

The assessment of overall nutrition remains challenging due to the multidimensional properties of grain foods. Key physiological determinants such as macronutrient composition (including protein and lipids), gut microbiome interaction, hormonal responses, and genetic variability may significantly affect glycemic index (GI) and were not examined. Besides grain texture, various grain components like bran, husk, kernel, and endosperm, and the differences between germinating and non-germinating forms, as well as size and shape, were not evaluated, despite their potential to independently influence GI. Future studies integrating these variables may improve statistical modeling and enhance the accuracy of glycemic predictions.

Another limitation is the lack of comprehensive carbohydrate quality metrics such as carbohydrate-to-fiber ratios and free sugar content, which are increasingly recognized as important indicators of dietary quality. Additionally, the incorporation of total and saturated fat, protein, and micronutrients may also support more robust classification of grain varieties with respect to GI and GL. Furthermore, in vivo comparisons of whole grains, their constituent parts, and refined counterparts are needed to clarify their distinct effects on glycemic control. Results from such investigations should be validated using clinically relevant outcomes within dietary patterns emphasizing low GI/GL. Existing evidence shows strong associations with glycated hemoglobin and HDL cholesterol, and moderate associations with body mass index, blood pressure, glycemia, apolipoprotein B, and C-reactive protein, highlighting the need for additional high-quality clinical trials. Collectively, these considerations underscore the necessity for future research evaluating health outcomes associated with whole versus refined grains and their fiber characteristics, to support more precise grain selection in diverse populations.

## Figures and Tables

**Table 1:** Composition of various carbohydrates (g), i.e., TC, TS, DS, and DF expressed per 100 g serving, along with the glycemic index (GI) and the glycemic load (GL), in various grains.

S.no.	Grains	Total Carbohydrate (TC)	Dietary Fiber (DF)	Dietary Starch (DS)	Total Sugar (TS)	Glycemic Index (GI)	Glycemic Load (GL)
1	Amaranth	68.8	27.34	55.7	1.7	70	43.4
2	Barley	77.4	16.2	56.9	0.8	70	67
3	Buck wheat (Whole)	71.1	4.8	61.6	2.6	50	22
4	Bulgur (Raw, Dry)	75.9	11.7	62.3	0.4	55	10.4
5	Black Bean	19.8	6.69	11.8	2.1	30	2
6	Bean Cannellini	18.8	6.76	11.3	0	35	21.4
7	Bread (White)	49.2	2.3	37.2	5.34	75	11
8	Bread wheat (Whole)	43.1	6	28.7	4.41	70	19
9	Chia seed	42	34	0.8	8	30	12.6
10	Chickpeas	20.3	5.92	12.8	11	36	9
11	Corn flour (Yellow)	80.8	4.3	67.3	1.04	65	10.1
12	Corn flour (White)	76.7	7	67.3	0.6	55	10.4
13	Corn (Sweet)	14.7	2.4	4.08	7.37	55	10.4
14	Einkorn (Dry, Raw)	68.6	8.9	56.2	0	45	8.1
15	Farro	72.1	7.3	61.7	2.2	40	11.3
16	Flaxseed	34.4	23.1	1.3	1.55	35	0.6
17	Almond flour	16.2	9.3	0	7.1	20	15.1
18	Barley flour	77.4	16.2	56.9	0.8	30	16.8
19	Buckwheat flour	56.3	5	54.9	1.4	40	28.2
20	Cassava flour	87.3	7.66	79.7	2	55	20.9
21	Chestnut flour	80.4	16.2	47.32	26.5	65	46.1
22	Coconut flour	58.9	34.2	0	5.2	32	18.1
23	Corn flour (Yellow)	80.8	4.3	54.1	1.04	70	53.8
24	Oat flour	69.9	12.9	53.4	0.8	25	3
25	Potato flour	79.9	16.6	62.7	3.5	95	78.9
26	Quinoa flour	69.5	6.95	58.1	1.8	40	22.9
27	Rice flour (brown)	75.5	7.3	25	1	66	32
28	Rice flour (glutinous free)	72.4	2	72.2	0.7	95	76.1
29	Rice flour (white)	79.8	0.5	83.7	0.2	95	76.1
30	Rye flour	77.2	17.9	54.1	2.3	45	28.9
31	Semolina flour	73.8	3.2	74	2.4	66	14.7
32	Sorghum flour	77.4	8.16	66.3	1.9	70	46.5
33	Soy flour	18.7	18	12.3	6.4	25	4.5
34	Soy flour (fatted)	35.2	16	5.3	9.3	25	4.5
35	Spelt flour	66.2	4	65.9	0.3	63	28
36	Wheat flour (all purpose)	73.2	2.72	58	0.24	85	62.6
37	Fonio	81.3	2.2	78.2	0	57	17.5
38	Kidney beans (Red)	21.5	5.4	36.7	3.85	23	6
39	Lentils	63.54	10.93	37.1	2.083	22	3
40	Millet	74.4	3.33	67.1	1.7	70	51.1
41	Oat	69.9	12.9	53.4	1.5	58	16
42	Quinoa flour	69.5	6.95	58.1	1.8	53	9
43	Rice (Black)	77.2	4.2	71.4	0	45	33.8
44	Rice (Brown)	76.7	4.3	71.6	0.7	55	18
45	Rice (Red)	76.2	4.2	70.8	0	55	38.8
46	Rice (White)	80.3	2.77	74.4	0.1	64	26
47	Sorghum bran	68.7	35	32.7	1	70	46.5
48	Sorghum flour (white)	73.5	3.3	69.7	1.9	70	46.5
49	Sorghum (White)	74.9	3.9	70.7	2.5	70	46.5
50	Sorghum (Whole grain)	73.6	8.3	65.6	2.5	70	46.5
51	Sunflower Seed	24.5	7.2	1	3.4	35	7
52	Rice (Wild)	75.7	4.3	68.6	2.5	35	7.3

Note: The data presented are mean values derived from published sources or provided as a single value from a database. The whole grain and whole grain flour values are presented separately.

**Table 2:** Estimated values of carbohydrate content-to-fiber ratio and net carbohydrate content in various grains.

S.no.	Grains	Carbohydrate-to-Fiber Ratio			Available Carbohydrate		
		Total Carbohydrate (TC/DF)	Total Sugar (TS/DF)	Dietary Starch (DS/DF)	Total Carbohydrate (TC-DF)	Total Sugar (TS-DF)	Dietary Starch (DS-DF)
1	Amaranth	2.516	0.0622	0.810	41.460	−25.64	28.360
2	Barley (Flour)	4.778	0.0494	0.735	61.200	−15.40	40.700
3	Buck wheat whole grain	14.813	0.5417	0.866	66.300	−2.20	56.800
4	Bulgur (Raw, Dry)	6.487	0.0342	0.821	64.200	−11.30	50.600
5	Black Bean	2.960	0.3139	0.596	13.110	−4.59	5.110
6	Bean Cannellini	2.781	0.0000	0.601	12.040	−6.76	4.540
7	Bread (White)	21.391	2.3217	0.756	46.900	3.04	34.900
8	Bread (Whole wheat)	7.183	0.7350	0.666	37.100	−1.59	22.700
9	Chia seed	1.235	0.2353	0.019	8.000	−26.00	−33.200
10	Chickpeas	3.429	1.8581	0.631	14.380	5.08	6.880
11	Corn flour (Yellow)	18.791	0.2419	0.833	76.500	−3.26	63.000
12	Corn flour (White)	10.957	0.0857	0.877	69.700	−6.40	60.300
13	Corn (Sweet)	6.125	3.0708	0.278	12.300	4.97	1.680
14	Einkorn (Dry, Raw)	7.708	0.0000	0.819	59.700	−8.90	47.300
15	Farro	9.877	0.3014	0.856	64.800	−5.10	54.400
16	Flaxseed	1.489	0.0671	0.380	11.30	−21.55	−21.80
17	Almond (Flour)	1.742	0.7634	0.000	6.900	−2.20	−9.300
18	Barley (Flour)	4.778	0.0494	0.735	61.200	−15.40	40.700
19	Buckwheat (Flour)	11.260	0.2800	0.975	51.300	−3.60	49.900
20	Cassava (Flour)	11.397	0.2611	0.913	79.640	−5.66	72.040
21	Chestnut (Flour)	4.963	1.6358	0.589	64.200	10.30	31.120
22	Coconut (Flour)	1.722	0.1520	0.000	24.700	−29.00	−34.200
23	Corn (Flour-Yellow)	18.791	0.2419	0.670	76.500	−3.26	49.800
24	Oat (Flour)	5.419	0.0620	0.764	57.000	−12.10	40.500
25	Potato (Flour)	4.813	0.2108	0.785	63.300	−13.10	46.100
26	Quinoa (Flour)	10.000	0.2590	0.836	62.550	−5.15	51.150
27	Rice (Flour-brown)	10.342	0.1370	0.331	68.200	−6.30	17.700
28	Rice (Flour-glutinous free)	36.200	0.3500	0.997	70.400	−1.30	70.200
29	Rice (Flour white)	159.600	0.4000	1.049	79.300	−0.30	83.200
30	Rye (Flour)	4.313	0.1285	0.701	59.300	−15.60	36.200
31	Semolina (Flour)	23.063	0.7500	1.003	70.600	−0.80	70.800
32	Sorghum (Flour)	9.485	0.2328	0.857	69.240	−6.26	58.140
33	Soy (Flour)	1.039	0.3556	0.658	0.700	−11.60	−5.700
34	Soy (Flour fatted)	2.20	0.5813	0.151	19.20	−6.70	−10.700
35	Spelt (Flour)	16.550	0.0750	0.995	62.200	−3.70	61.900
36	Wheat all purpose (Flour)	26.912	0.0882	0.792	70.480	−2.48	55.280
37	Fonio	36.955	0.0000	0.962	79.100	−2.20	76.000
38	Kidney beans (Red)	3.981	0.7130	1.707	16.100	−1.55	31.300
39	Lentils	5.813	0.1906	0.584	52.610	−8.85	26.170
40	Millet	22.342	0.5105	0.902	71.070	−1.63	63.770
41	Oat	5.419	0.1163	0.764	57.000	−11.40	40.500
42	Quinoa (Flour)	10.000	0.2590	0.836	62.550	−5.15	51.150
43	Rice (Black)	18.381	0.0000	0.925	73.000	−4.20	67.200
44	Rice (Brown)	17.837	0.1628	0.934	72.400	−3.60	67.300
45	Rice (Red)	18.143	0.0000	0.929	72.000	−4.20	66.600
46	Rice (White)	28.989	0.0361	0.927	77.530	−2.67	71.630
47	Sorghum bran	1.963	0.0286	0.476	33.700	−34.00	−2.300
48	Sorghum (Flour white)	22.273	0.5758	0.948	70.200	−1.40	66.400
49	Sorghum (White)	19.205	0.6410	0.944	71.000	−1.40	66.800
50	Sorghum (Whole grain)	8.867	0.3012	0.891	65.300	−5.80	57.300
51	Sunflower Seed	3.403	0.4722	0.041	17.3	−3.80	−6.20
52	Rice (Wild)	17.605	0.5814	0.906	71.400	−1.80	64.300

Note: The values presented in the tables are approximate carbohydrate content per 100 g serving in various grains. Available (net) carbohydrate content was determined in various grains by subtracting the total fiber content from the total carbohydrate content, dietary starch, and total sugar.

**Table 3:** Statistical results of PCA of various components in grains (*n* = 52).

PC Summary	PC1	PC2	PC3	PC4
Eigenvalue	2.291	0.8830	0.7591	0.06726
Proportion of variance	57.27%	22.08%	18.98%	1.68%
Cumulative proportion of variance	57.27%	79.34%	98.32%	100.00%

**Table 4:** MLRA between carbohydrate content and carbohydrate-to-dietary fiber ratios with GI across various grains (*n* = 52).

	Grains Carbohydrate				Carbohydrate Content to Fiber Ratio		
Values	Total Carbohydrates	Total Sugar	Dietary Starch	Dietary Fiber	Total Carbohydrates	Total Sugar	Dietary Starch
R	0.5625	0.5601	−0.1553	−0.2650	0.4833	0.0444	0.3330
R^2^	0.3164	0.3137	0.0241	0.0702	0.2336	0.00197	0.1109
*p*-values	<0.0001	<0.0001	0.2715	0.0577	0.0003	0.7544	0.0159
Significance	****	****	ns	ns	***	ns	*

Note: Asterisks indicate levels of statistical significance “****” *p* < 0.0001 (highly significant), “***” *p* < 0.0005 (moderately significant), “*” *p* < 0.05 (low significance), ns = not significant.

**Table 5:** MLRA between available (net) carbohydrate content and available carbohydrate content-to-DF ratios with GI across various grains (*n* = 52).

	Available Carbohydrate			Available Carbohydrate-to-Dietary Fiber		
Values	Total Carbohydrates	Total Sugar	Dietary Starch	Total Carbohydrates	Total Sugar	Dietary Starch
R	0.5926	0.1879	0.5420	0.4833	0.04445	0.4566
R^2^	0.3512	0.03531	0.2938	0.2336	0.001975	0.2085
*p*-values	<0.0001	0.1822	<0.0001	0.0003	0.7544	0.0007
Significance	****	ns	****	***	ns	***

Note: Asterisks indicate levels of statistical significance “****” *p* < 0.0001 (highly significant), “***” *p* < 0.0005 (moderately significant), ns = not significant.

**Table 6:** Pearson’s regression analysis of carbohydrate content and carbohydrate content-to-DF ratios with GI.

	Grains Carbohydrates			Carbohydrates Content-to-Fiber Ratio		
Values	TC	TS	DS	TC	TS	DS
R	0.5625	−0.1553	0.5601	0.4833	0.04444	0.3330
R^2^	0.3164	0.02413	0.3137	0.2336	0.001975	0.1109
*p*-values	<0.0001	0.2715	<0.0001	0.0003	0.7544	0.0159

**Table 7:** Pearson’s regression analysis of net available carbohydrate content and available carbohydrate content-to-DF ratios with GI.

	Net Grains Carbohydrates			Net Carbohydrates Content-to-Fiber Ratio		
Values	TC	TS	DS	TC	TS	DS
R	0.5926	0.1879	0.5420	0.4833	0.04445	0.4566
R2	0.3512	0.03531	0.2938	0.2336	0.001975	0.2085
*p*-values	<0.0001	0.1822	<0.0001	0.0003	0.7544	0.0007

## Data Availability

The original contributions presented in the study are included in the article/Supplementary Materials; further inquiries will be made available on request.

## Supplementary Materials

The following supporting information can be downloaded at: https://www.mdpi.com/article/10.3390/foods14234038/s1, Figure S1: (A–D): Multiple linear regression analysis (MLRA) examining the relationship between glycemic load and various grain (n = 52) carbohydrate contents. (A). MLRA of TC, TS, DS, and DF in relation to GL. (B). MLRA between grains content-to-DF ratio and GL. (C). MLRA between available (net) content and GL. (D). MLRA between available content-to-DF ratios and GL. In all plots, each data point represents an individual grain sample, with carbohydrate content values plotted on the *Y*-axis and GL on the *X*-axis. Solid lines indicate the fitted regression models, illustrating the strength and direction of the associations; Figure S2: (A–F): Correlation plots illustrating the correlation between the GL and both carbohydrates content and carbohydrates-to-DF ratios in various grains (n = 52). (A–C) display correlations between GL and individual carbohydrate components. (D–F) show correlation between GL and the corresponding carbohydrates-to-DF ratios. Each data point represents a distinct grain sample, with the GL values plotted on the *Y*-axis and the grain contents on the *X*-axis. The solid lines represent linear regression trends, showing the direction and strength of the association across the grains’ samples; Figure S3: A-F: Correlation plot illustrating the relationship between the GL and the net carbohydrate content as well as net carbohydrate content-to-DF ratios in various grains (n = 52). (A–C) display the correlation between individual available carbohydrate components. (D-F) display the correlation between GL and the corresponding net available (net) carbohydrate contents-to-DF ratios. Each data point represents a distinct grain sample, with GL values plotted on the *Y*-axis and net carbohydrate contents and net carbohydrate -to-DF ratios on the *X*-axis. Solid lines represent linear regression trends, showing the direction and strength of the associations across the grains’ samples; Table S1: Multiple linear regression analysis between carbohydrate content and carbohydrates-to-DF ratios with GL across various grains (n = 52); Table S2: Multiple linear regression analysis between available carbohydrates contents and available carbohydrate content-to-DF ratios with GL across various grains (n = 52); Table S3: Pearson’s regression analysis of carbohydrate content and carbohydrate content-to-DF ratios with GL; Table S4: Pearson’s regression analysis of net available carbohydrate content and available carbohydrate content-to-DF ratios with GL.

## Abbreviations

The following abbreviations are used in this manuscript:

AUC	Area under the curve
IBS	Irritable bowel syndrome
CGM	Continuous glucose monitoring
CRP	C-reactive Protein
CVD	Cardiovascular disease
CKD	Chronic Kidney disease
DS	Dietary Starch
DF	Dietary fiber
DGTAC	Dietary guidelines technical advisory committee
DGA	Dietary guidelines for Americans
FDA	Food and drug administration
FGM/isCGM	Flash/Intermittently scanned continuous glucose monitoring
GI	Glycemic index
GL	Glycemic load
GR	Glycemic response
GLP-1	Glucagon-like peptide
HbA1c	Hemoglobin A1c
HHS	U.S. Department of Health and Human Services
iAUC	Incremental area under the curve
IDF	Insoluble dietary fiber
KFCD	Korean Food Composition Database
LDL	Low density lipoprotein
LE	Life expectancy
MLRA	Multiple linear regression analysis
NAFLD	Non-alcoholic fatty liver disease
NSP	Non-starch Polysaccharides
OS	Oligosaccharides
PCA	Principle component analysis
RG	Refined Grain
RS	Resistant starch
ROS	Reactive oxygen species
RT-CGM	Real-time Continuous glucose monitoring
SCFAs	Short-chain fatty acids
SDF	Soluble dietary fiber
T2DM	Type 2 Diabetes mellitus
TC	Total Carbohydrates
TS	Total Starch
WHO	World Health Organization
WG	Whole Grain
WGC	Whole grain Council
