# Supplementary material for: Grains, Cereals, and Legumes: Implications in Glycemic Index and Perspectives
Source: Foods. 2025 Nov 25;14(23):4038. doi: 10.3390/foods14234038 (PMC12691718; doi:10.3390/foods14234038)
Supplement: Supplementary file 1 [file foods-14-04038-s001.zip › foods-3928242-supplementary.pdf]

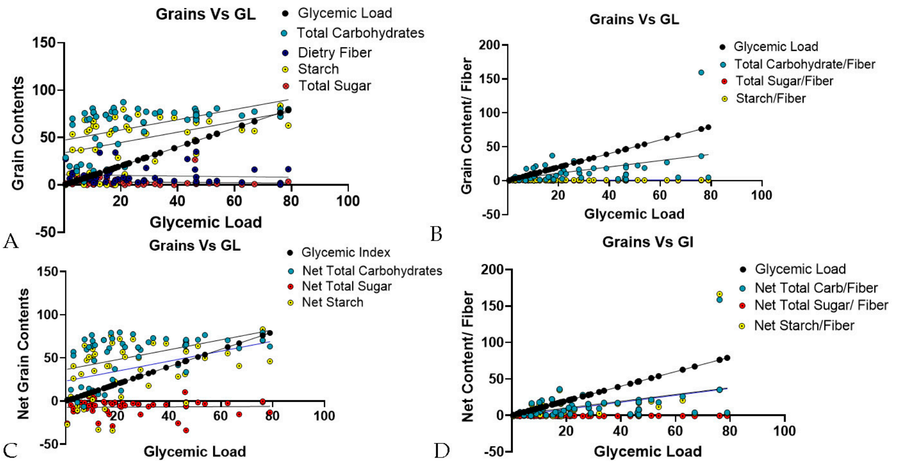

Figure S1. A-D: Multiple linear regression analysis (MLRA) examining the relationship between glycemic load and various grain (n=52) carbohydrate contents. **A**. MLRA of TC, TS, DS, and DF in relation to GL. **B**. MLRA between grains content-to-DF ratio and GL. **C**. MLRA between available (net) content and GL. **D**. MLRA between available content-to-DF ratios and GL. In all plots, each data point represents an individual grain sample, with carbohydrate content values plotted on the Y-axis and GL on the X-axis. Solid lines indicate the fitted regression models, illustrating the strength and direction of the associations.

Table S1. Multiple linear regression analysis between carbohydrate content and carbohydrates-to-DF ratios with GL across various grains (n=52).

	Grains carbohydrates				Carbohydrates content to fiber ratio		
Values	TC	TS	DS	DF	TC	TS	DS
R	0.4906	0.4493	-0.07954	-0.07954	0.4379	-0.1387	0.2488
R2	0.2407	0.2019	0.006326	0.006326	0.1918	0.01925	0.06192
p-values	0.0002	0.0008	0.5751	0.5751	0.0012	0.3267	0.0753
Significance	***	***	ns	ns	**	ns	ns

Table S2. Multiple linear regression analysis between available carbohydrates contents and available carbohydrate content-to-DF ratios with GL across various grains (n=52).

	Available carbohydrates			Available carbohydrate-to-DF ratio		
Values	TC	TS	DS	TC	TS	DS
R	0.4669	0.03463	0.3977	0.4379	-0.1387	0.4266
R2	0.2180	0.001199	0.1582	0.1918	0.01925	0.1820
p-values	0.0005	0.8074	0.0035	0.0012	0.3267	0.0016
Significance	***	ns	**	**	ns	**

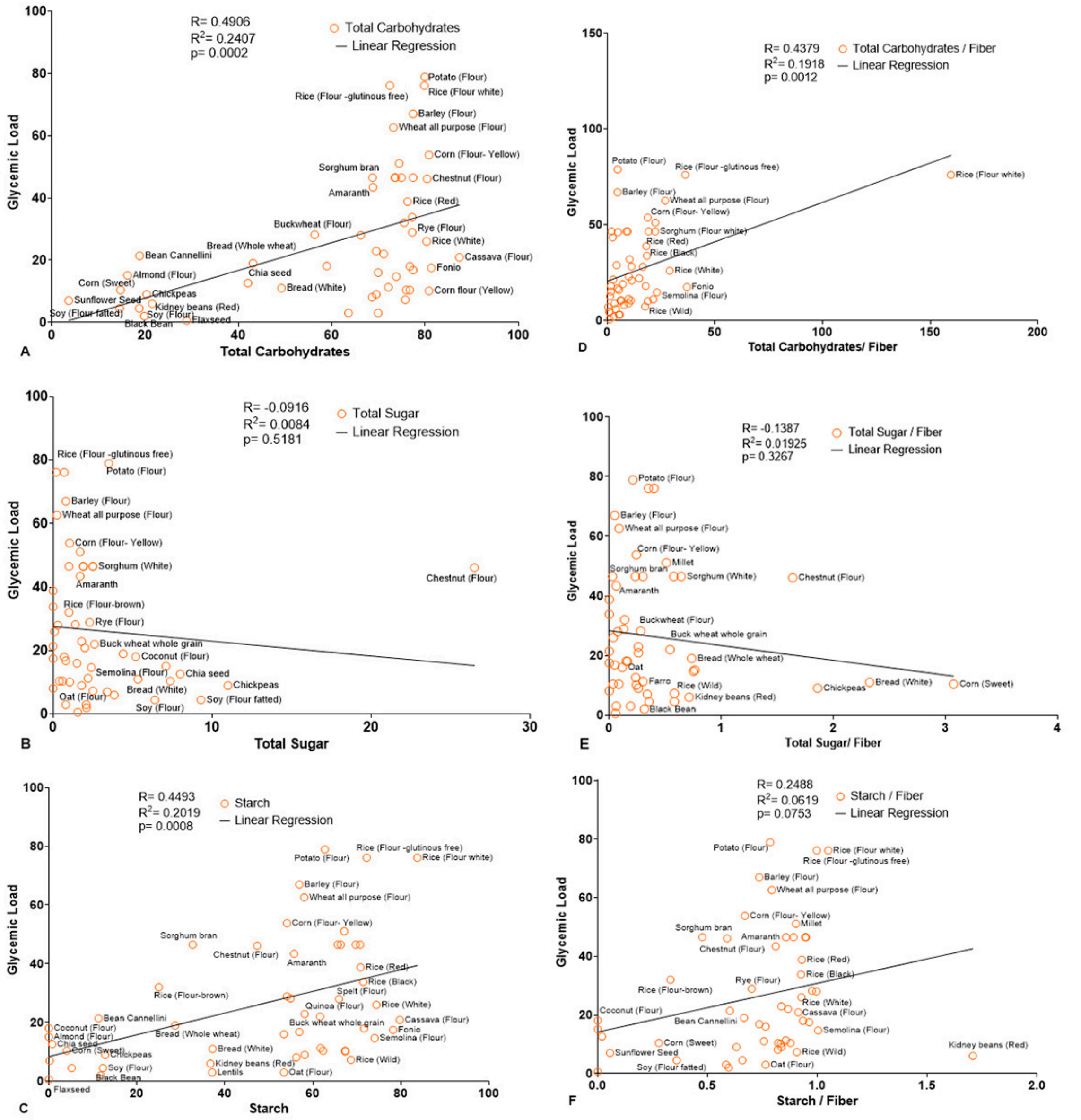

Figure S2. A-F: Correlation plots illustrating the correlation between the GL and both carbohydrates content and carbohydrates-to-DF ratios in various grains (n=52). **(A-C)** display correlations between GL and individual carbohydrate components. **(D-F)** show correlation between GL and the corresponding carbohydrates-to-DF ratios. Each data point represents a distinct grain sample, with the GL values plotted on the Y-axis and the grain contents on the X-axis. The solid lines represent linear regression trends, showing the direction and strength of the association across the grains' samples.

Table S3. Pearson's regression analysis of carbohydrate content and carbohydrate content-to-DF ratios with GL.

	Grains Carbohydrates			Carbohydrates content to fiber ratio		
Values	TC	TS	DS	TC	TS	DS
R	0.4906	-0.09166	0.4493	0.4379	-0.1387	0.2488
R2	0.2407	0.00840	0.2019	0.1918	0.01925	0.06192
p-values	0.0002	0.5181	0.0008	0.0012	0.3267	0.0753

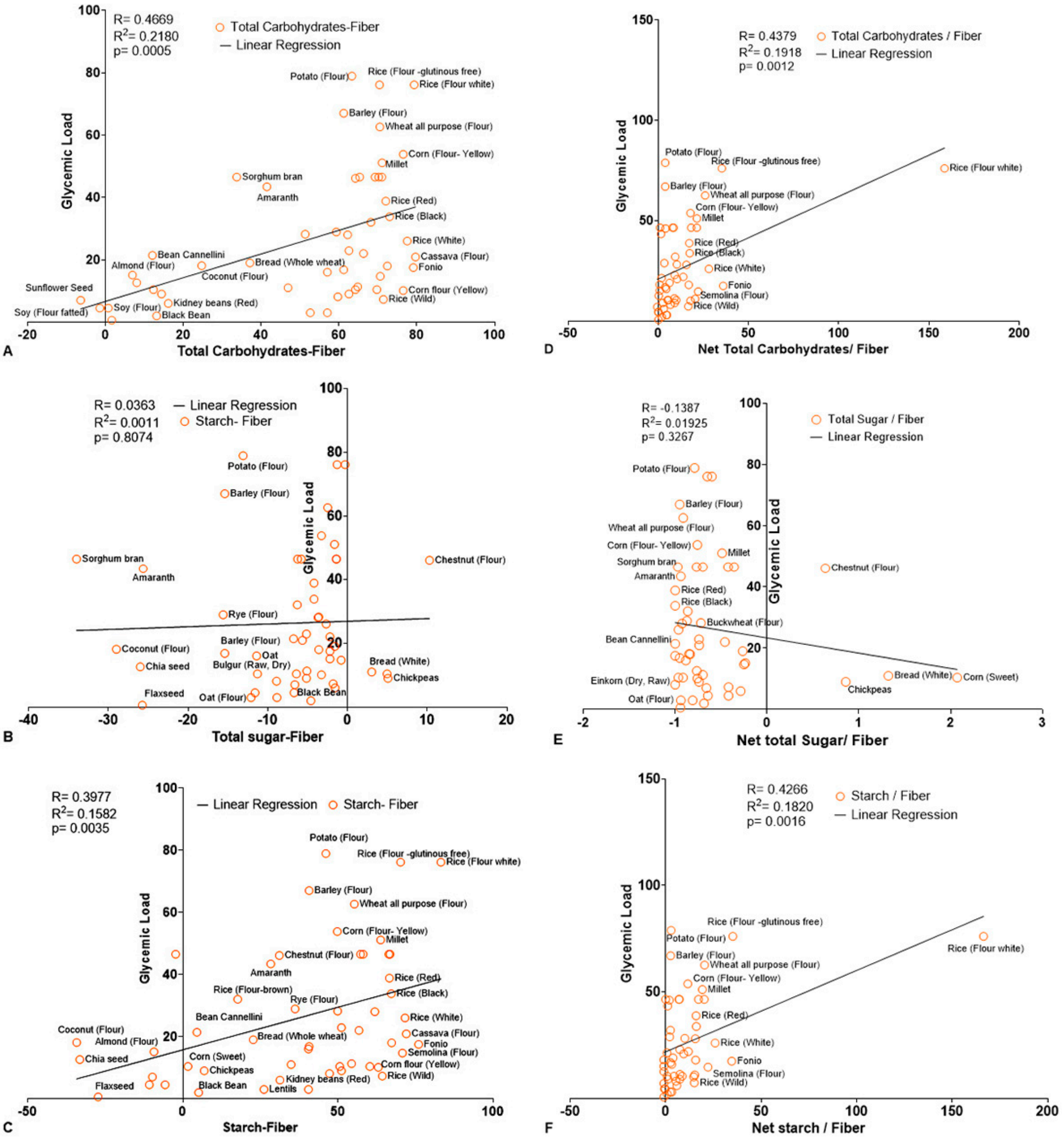

Figure S3. A-F: Correlation plot illustrating the relationship between the GL and the net carbohydrate content as well as net carbohydrate content-to-DF ratios in various grains (n=52). **(A-C)** display the correlation between individual available carbohydrate components. **(D-F)** display the correlation between GL and the corresponding net available (net) carbohydrate contents-to-DF ratios. Each data point represents a distinct grain sample, with GL values plotted on the Y-axis and net carbohydrate contents and net carbohydrate -to-DF ratios on the X-axis. Solid lines represent linear regression trends, showing the direction and strength of the associations across the grains' samples.

Table S4. Pearson's regression analysis of net available carbohydrate content and available carbohydrate content-to-DF ratios with GL.

	Net grains carbohydrates			Net carbohydrates content to fiber ratio		
Values	TC	TS	DS	TC	TS	DS
R	0.4669	0.0363	0.3977	0.4379	-0.1387	0.4266
R2	0.2180	0.001199	0.1582	0.1918	0.01925	0.1820

p-values	0.0005	0.8074	0.0035	0.0012	0.3267	0.0016
